# Cure of Club-Foot, Bent-Knee, Wry-Neck, Spinal and Other Deformities

**Published:** 1840-01

**Authors:** 


### ART. X.

1. *Cure of Club-foot, Bent-knee, Wry-neck, Spinal and other Deformities. With Remarks on the late progress of Art, and on the necessity of a Public Institution for the Relief of the Poor, labouring under Deformities.* By GUSTAV KRAUSS, M.D., Member of several learned Societies. With Cases and Woodcuts.—*London*, 1839. 8vo, pp. 42.

2. *Beiträge zur subcutanen Orthopädie, oder über die Heilung angeborner oder erworbener Contracturen der Glieder mittelst Durchschneidung der verkürzten Sehnen und Muskeln unter der Haut.* Mitgetheilt von Geh. Med. Rath Prof. Dr. DIEFFENBACH, in Berlin.

*Contributions to the subject of Subcutaneous Orthopædia, or the Cure of congenital or acquired Contractions of Joints, by division of the shortened tendons and muscles under the skin.* Communicated by Dr. DIEFFENBACH, of Berlin, to the "Wochenschrift für die gesammte Heilkunde." Nos. for September 21 and 28.—*Berlin*, 1839.

As we have so recently presented our readers with a detailed account of the principles and practice of operative Orthopædia (see our last No.), a brief notice of the above additions to our stock of information is all that will be needed: and this may be regarded as an appendix to our former article.

First, with regard to Dr. Krauss's pamphlet: after a careful examination of its contents, and consideration of its pretensions, we are sorry to express our decided disapprobation of a work which, it is too palpable, would never have seen the light, but for the hope of some personal advantage accruing therefrom to the author. We are the more reluctant to appear thus harsh in our criticism, as the author is a foreigner sojourning amongst us; and because we are far from expecting that the time has arrived when the only really justifiable motive to professional men for becoming authors, holds a solitary sway,—we mean the well-grounded anticipation of adding to the general stock of information: but when an author appears before us without any novelty to communicate, whose book is full of reclamations and self-extolling paragraphs, and this under the cloak of purely disinterested, humane, and scientific motives, we do feel ourselves called upon to ask, at least, whether there can be here any thing like the prostitution of good ostensible objects to a really selfish end? This question refers chiefly to the appeal which Dr. Krauss makes, in the form of an appendix to his pamphlet, to the humane public in behalf of the afflicted poor suffering from deformities. Would it, we would further ask, be any blow to the doctor's disinterestedness, were such an institution as he desires, to be established without his being appointed one of the medical officers? A few brief quotations from the author's preface will account for our doubts respecting this publication: "In offering these pages to the public (says the author), I obey the dictates of my heart, and discharge a duty which I feel due to those of my poorer patients, whose misfortune it is to labour under deformities. A tour for purposes purely scientific brought me to England in the year 1837. . . . . With zeal and delight I continued my labours. Obeying the calls of the afflicted, I penetrated the remotest districts of London, unremittingly seeking to bring to perfection the powers of art in the cure of deformities. . . . . The best proofs of my success are the cures I have effected, particularly those of congenital club-foot, of the highest degree, and in patients of an advanced age. It is far from my intention to exalt my own merits, or cast blame on others for not having been equally successful. The cause of my success is attributable to my having dedicated myself more to this subject, and taken peculiar pleasure in its study. Shrinking from no sacrifice where the welfare of my patients and the development of science were concerned, I proceeded steadily in my career," &c. &c. Dr. Krauss presumes to lay down as an axiom that "there may be excellent surgeons who are, nevertheless, no orthopædists:" and that "institutions for the cure of deformities ought to be under the superintendence of men who have particularly dedicated themselves to the subject." Now, *we* presume to deprecate *in toto* this exclusive system. What! we would ask, is the noble discovery of which we treat—a scientific deduction from just premises by a scientific surgeon—to degenerate into a mechanical art, and be placed in the hands of the mechanic? Are not Stromeyer and Dieffenbach surgeons of whom Dr. Krauss's country has reason to be proud? We repeat, as we observed in our former article and in spite of all Dr. Krauss's egotism and vanity, that vast numbers of our surgeons are as capable of treating deformities, and have been as successful in the results obtained, as himself or any other exclusive orthopædist.

In concluding this our notice of Dr. Krauss's pamphlet, we must do him the justice to say that the cases he relates show that he is acquainted with the principles of his subject, and has been a successful operator.

We now turn to the more grateful task of laying before our readers a summary of Prof. Dieffenbach's vast experience, which he—mark the contrast—is satisfied to present to the public in the form of a paper communicated to a weekly journal:—not for want of materials to build a book,—but doubtless because he appreciates the merits of those already published, and is conscious that the only additions called for are the *general* results of extended experience. In the following paragraphs we shall communicate all the principal results recorded in the Professor's Memoir, and with little or no comment of our own; and although they are not either a close or continuous translation of the original, we shall give them the ordinary form of translation:

"In the case of contracted joints, the division of a tendon does not in itself constitute the cure, but is only a necessary preparatory step to placing the limb in a condition by which the cure may be afterwards effected. Many cases which I had deemed cured by mechanical means alone, have presented themselves again to me with a return of the deformity.

"I have now operated on about three hundred club-feet, and sixty wry-necks: independently of which, a great number of contracted arms, fingers, hip and knee joints, feet and toes have been likewise cured by operation, under my care. In all cases the contracted tendons have been divided with the aid of a small, sickle-shaped knife, similar to the old penknives, though smaller. Neither dangerous hemorrhage nor division of any important vessel ever took place; but a drop of blood was usually all that followed the division of the achilles tendon or the sterno-mastoid muscle; or, where more bleeding by chance followed, it was always arrested by the application of a bandage. Where suppuration ensued, which was rare, a small dilatation of the opening so as to allow the free escape of the matter was generally sufficient to bring about the cure. Pressure of the apparatus on the outer border or dorsum of the foot not unfrequently produced troublesome swelling, and occasioned the necessity of intermitting the use of the mechanical means for a time. Symptoms of nervous irritation, whether mild or acute, whether trismus or tetanus, never once exhibited themselves; and death was in no instance, whether as an immediate effect or secondary result, the consequence of the operation. In six or eight cases only was the operation for club-foot unsuccessful; and in some of these the want of success was attributable to the carelessness or fractiousness of the patient; whilst others submitted a second time to the operation, and were then cured. Age made no difference in the results. The youngest child on whom I operated was but three days old, whilst the eldest was fifty-four. [We presume the former was an experiment, and scarcely to be recommended as a precedent.] In *wry-neck,* sometimes the sternal, sometimes the acromial portion was divided near to the insertion: and many of these patients had for a long time vainly employed mechanical means. In one case of this class, a curious optical phenomenon was observed, viz., that immediately after the division of the muscle, and return of the head suddenly to its normal position, the patient exclaimed, 'I see all things awry.'

"In simple *pes equinus,* the plan pursued was, to divide the tendo achillis about two inches above the heel, and then cover the orifice with a broad band of adhesive plaster; and if the blood ceased to ooze, in two or three days, Stromeyer's apparatus was applied, and the cases usually did well in a few weeks. But in extreme cases and irritable subjects, the following plan was adopted: the limb was carefully and evenly bound with a roller from the toes to the calf, and the bandage then saturated with boiled starch, or a solution of resin in alcohol. I then directed the patient to stand upright, and press the bound-up foot firmly on the ground, and remain in that position till the bandage had become stiff and dry; and this answered the purpose of an apparatus. This proceeding was repeated twice a-week until the cure was completed.

"In the *pes varus,* the division of the achilles tendon, with the after-aid of adhesive plaster, or a bandage prepared as above, or with thin plaster of paris, were in general sufficient to procure a successful result in young children; but Scarpa's boot or Stromeyer's apparatus were found necessary in cases of longer standing and in older subjects. The various tendons divided for this form of distortion were those of achilles, tibialis anticus, flexor pollicis longus, or the plantar fascia. If the case proved obstinate, the tendons were frequently divided again and again; thus the achilles tendon was cut two, three, or four times before the cure was complete. In one adult with enormous club-feet of the highest degree, those divisions were repeated twenty times before a successful termination was obtained. [A pretty good evidence of the reproductive power of tendinous fibre or its substitute.] In extreme cases of club-foot, it is requisite to divide all the opposing flexor and extensor tendons in turn: the division of the achilles tendon should then be undertaken last of all.

"In *flat* or *splay-foot,* I have in all cases which have come under my care, whether of children or adults, divided all the long extensors on the dorsum of the foot: plaster and bandage were then applied to the hanging foot, and a straight splint placed on the anterior surface of the leg, extending to the dorsum of the foot, so as to keep it in the position of pes equinus, a matter of no difficulty, when the extensor resistance was removed: the result of this treatment was equally successful with that which followed division of the flexor tendons in the other two forms above described. [This treatment is peculiar, and differs widely from the simpler treatment of Stromeyer,—see our last Number, p. 399. One would scarcely have judged *à priori,* that the arch of the foot would have been restored by dividing the extensor tendons—so much, however, for theory, as opposed to actual experience.] The same treatment is applicable in spasmodic and intermittent cases.

"The cases which required the division of the hamstring muscles were conducted in the following manner: either two or three punctures were made, according to circumstances, the same little sickle-knife being employed: the limb was then extended, and a broad strip of plaster placed around it; the position being in some measure preserved by the aid of a compress or pad, and pasteboard splint: and when all inflammatory action had ceased, Stromeyer's apparatus was applied. I have also employed a more speedy mode of obtaining the desired position of the limb. Having divided the proper tendons, I flexed the leg on the thigh so as to approximate the heel and buttock, and then suddenly extended the limb with sufficient force to bring it at once straight: this action was accompanied with a loud cracking noise, as if something had given way. A bandage of flannel (on account of its elasticity) was then applied along the whole limb, and a long, light, padded splint placed along its posterior aspect, and retained in position by appropriate strips of linen.

"In bent-arm, after division of the biceps tendon, the extremity has been forthwith extended, and the cure thus effected in a fortnight. In bent fingers, resulting from rheumatic or other affections about the joints, the success depends upon rendering them moveable as well as straight; and in this I have been very successful in relieving and curing those who would otherwise have sacrificed the fingers to get rid of the nuisance, for such it is amongst the working classes. In bent toes, the treatment is the same, though they are more resisting and troublesome. In the worst cases, division of both flexors and extensors was found requisite.

"I have often reduced dislocations of long standing, which were only rendered reducible by division of shortened tendons: as, for instance, that of the great pectoral muscle in dislocation of the humerus: and likewise I have divided the tendon of the biceps in long-standing luxation of the fore-arm; or those of the flexor carpi radialis and ulnaris, in dislocation of the hand. In one long-standing case of dislocation of the foot forwards upon the tarsus, I was enabled, by division of the tendo achillis, to reduce the limb to its normal position. [These cases must be regarded as most interesting and valuable, whether we consider them as evidences of the bold practice of the operator, or in the light of precedents for future practice.] In spasmodic contractions of the upper extremities resulting from affection of the brain, considerable relief has likewise been obtained by division of the biceps and other tendons."

---

